# Molecular properties of linear amino acids in water

**DOI:** 10.1007/s00726-023-03365-3

**Published:** 2024-02-01

**Authors:** Roman Boča, Richard Imrich, Juraj Štofko, Beáta Vranovičová, Cyril Rajnák

**Affiliations:** 1 Faculty of Health Sciences, University of SS Cyril and Methodius, 91701 Trnava, Slovakia; 2 Faculty of Natural Sciences, University of SS Cyril and Methodius, 91701 Trnava, Slovakia

**Keywords:** Glycine, β-Alanine, GABA, δ-Aminovaleric acid, Ab initio calculations, DFT calculations, Molecular descriptors

## Abstract

Four linear amino acids of increased separation of the carboxyl and amino groups, namely glycine (aminoacetic acid), β-alanine (3-aminopropanoic acid), GABA (4-aminobutanoic acid) and DAVA (5-aminopentanoic acid), have been studied by quantum chemical ab initio and DFT methods including the solvent effect in order to get electronic structure and molecular descriptors, such as ionisation energy, electron affinity, molecular electronegativity, chemical hardness, electrophilicity index, dipole moment, quadrupole moment and dipole polarizability. Thermodynamic functions (zero-point energy, inner energy, enthalpy, entropy, and the Gibbs energy) were evaluated after the complete vibrational analysis at the true energy minimum provided by the full geometry optimization. Reaction Gibbs energy allows evaluating the absolute redox potentials on reduction and/or oxidation. The non-local non-additive molecular descriptors were compared along the series showing which of them behave as extensive, varying in match with the molar mass and/or separation of the carboxyl and amino groups. Amino acidic forms and zwitterionic forms of the substances were studied in parallel in order to compare their relative stability and redox properties. In total, 24 species were investigated by B3LYP/def2-TZVPD method (M1) including neutral molecules, molecular cations and molecular anions. For comparison, MP2/def2-TZVPD method (M2) with full geometry optimization and vibrational analysis in water has been applied for 12 species; analogously, for 24 substances, DLPNO-CCSD(T)/aug-cc-pVTZ method (M3) has been applied in the geometry obtained by MP2 and/or B3LYP. It was found that the absolute oxidation potential correlates with the adiabatic ionisation energy; the absolute reduction potential correlates with the adiabatic electron affinity and the electrophilicity index. In order to validate the used methodology with experimental vertical ionisation energies and vibrational spectrum obtained in gas phase, calculations were done also *in vacuo*.

**Supplementary Information:**

The online version contains supplementary material available at 10.1007/s00726-023-03365-3.

## Introduction

Amino acids form a numerous class of compounds that counts over 500 substances existing in nature. Of them, the most important are 22 proteinogenic alpha-amino acids occurring in the genetic code. Hereafter, we are focussing to a series of linear (catena) aliphatic amino acids starting from the simplest member–glycine, β-alanine, γ-aminobutyric acid and δ-aminovaleric acid.

Glycine (aminoacetic acid, C_2_H_5_NO_2_) is a white solid, solubility in water 25 g/100 cm^3^, octanol/water partition coefficient log*P*_ow_ = − 3.21, acidity constants p*K*_a_ = 2.34 (carboxyl) and 9.60 (amino). β-alanine (3-aminopropanoic acid, C_3_H_7_NO_2_) is an isomer to the α-alanine; it is a white solid, solubility in water 54 g/100 cm^3^, log*P*_ow_ = − 3. 05, p*K*_a_ = 3.55 (carboxyl) and 10.24 (amino). GABA (4-aminobutanoic acid, γ-aminobutyric acid, C_4_H_9_NO_2_) is a white solid, solubility in water 150 g/100 cm^3^, log*P*_ow_ = − 3.17, p*K*_a_ = 4.02 (carboxyl) and 10.56 (amino). It contains 3 rotatable bonds. A continuation of this series is DAVA (δ-aminovaleric acid, 5-aminopentanoic acid, C_5_H_11_NO_2_), solubility in water 100 g/100 cm^3^, log*P*_ow_ = − 2.63, p*K*_a_ = 4.27, 4 rotatable bonds.

**Fig. 1:**
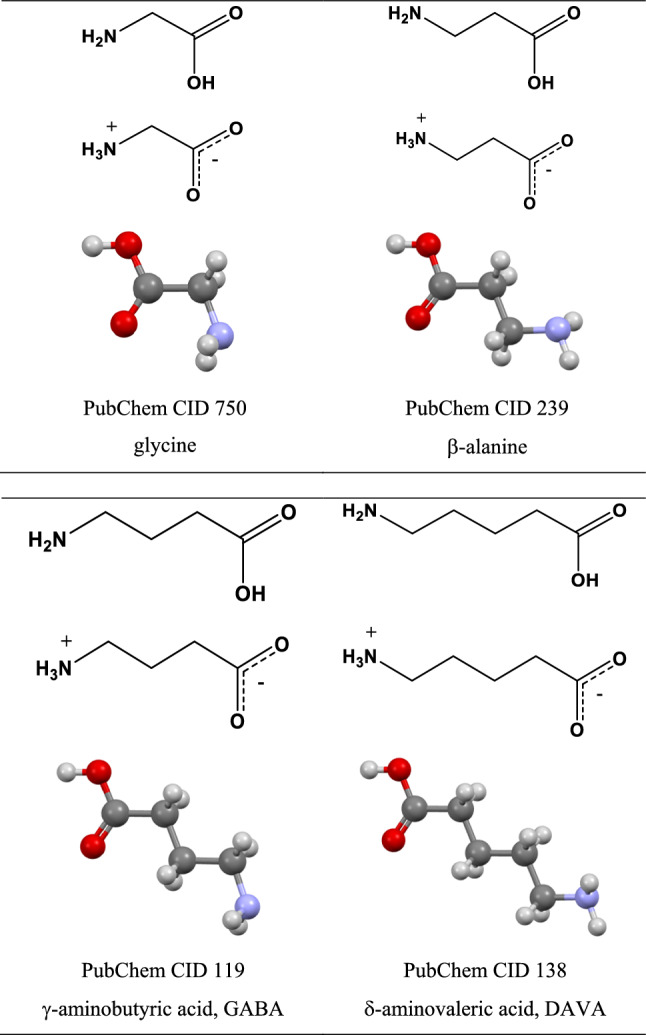
Structural formulas of linear amino acids including their zwitterionic forms. Colour code: white–H, black–C, blue–N, red–O

All species under study can exist in the canonical amino acid forms, or in the forms of zwitterions with spatially separated -NH_3_^+^ and -COO^−^ groups (Fig. 1). In solutions, complex equilibria between them depending upon pH exist. Solid-state zwitterionic forms are obtained by crystallisation from the neutral water solutions. The situation is much more complex: for instance, in the solid state, three anhydrous polymorphs of glycine were identified along with the dihydrate form (Drebushchak et al. 2002; Iitaka 1960, 1959, 1961; Jönsson and Kvick 1972; Kvick et al. 1980; Xu et al. 2017).

Glycine is one of the proteinogenic amino acids and is integral to the formation of alpha-helices in secondary protein structure due to its compact form. Glycine is the most abundant amino acid in collagen forming triple-helices. In the nervous system, glycine is also an inhibitory neurotransmitter. β-Alanine is a part of several key components of the intermediary metabolism, such as carnosine and of pantothenic acid (vitamin B5), which itself is a component of coenzyme A. The highest concentration of β-alanine is in skeletal muscles, heart and brain. Its pharmacological administration increases performance of muscle and reduces fatigue in athletes. GABA is the main inhibitory neurotransmitter in the developmentally mature mammalian central nervous system. Its principal role is reducing neuronal excitability throughout the nervous system (Boonstra et al. 2015). GABA deficiency is associated with several neurological and psychiatric diseases, such as epilepsy, schizophrenia and anxiety. DAVA is a delta-amino acid with a weak GABA agonist activity (Kristiansen et al. 1992). DAVA is also important in haem synthesis, and its increased level is significant in diagnosis of porphyria.

Amino acids are non-innocent with respect to redox processes. They reduce the iron(II) salts under anaerobic conditions to yellow nanoparticles of Fe(0) which, exposed to air, are readily oxidised to a mixture of dark-coloured iron oxides (Matelková et al. 2013; Klačanová et al. 2013; Vatrál et al. 2014).

An inspection of the databases (PubChem, DrugBank, HMDB—Human Metabolome Database) confirms that there a gap in the physicochemical properties of amino acids. That is our main motivation to fill this gap.

High-quality ab initio calculations that include a part of the correlation energy are very effective in studying the electronic structure and molecular properties of small bioactive molecules including amino acids (Bohórquez et al. 2003; Chipot et al. 1993; Flaig et al. 2002; Head-Gordon et al. 1997; Matta and Bader 2003; Matta 2014; Popelier and Aicken 2003). The aim of the present work is to calculate an extended set of non-local molecular descriptors associated with the electron transfer that involves the adiabatic ionisation energy, electron affinity, molecular electronegativity, chemical hardness and electrophilicity index using a consistent methodology. As these species are redox active, the absolute redox potentials on reduction and/or oxidation were enumerated based upon the reaction Gibbs energy. Which molecular descriptor correlates with the absolute reduction and oxidation potentials serves for a hypothesis. The non-local non-additive molecular descriptors were compared along the series showing which of them behave as extensive, varying in match with the molar mass and/or separation of the carboxyl and amino groups. In total, 24 species were investigated by B3LYP, 12 by MP2 and 24 by DLPNO-CCSD(T) methods including neutral molecules, molecular cations and molecular anions for canonical and zwitterion forms of linear amino acids in water as a solvent.

**Fig. 2:**
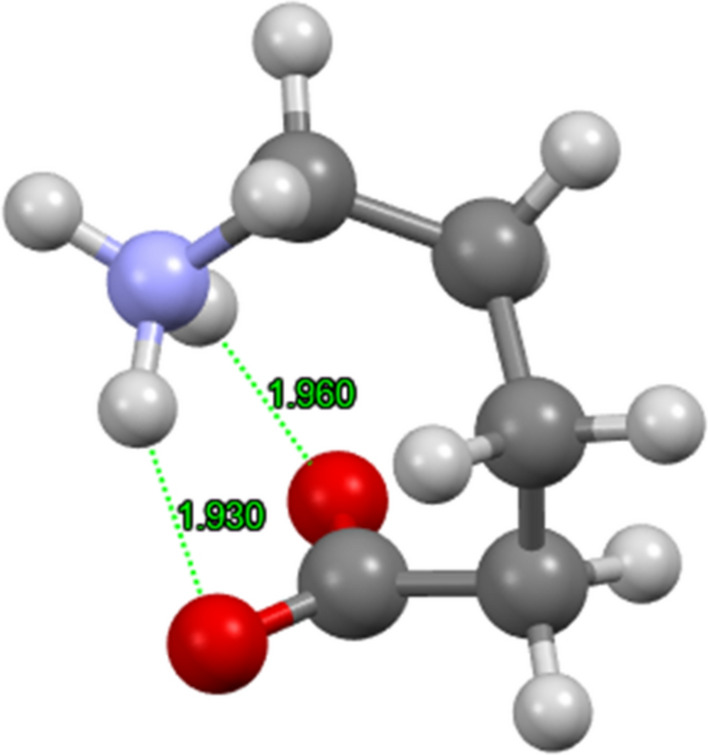
A wrapped structure of DAVA

There is an obstacle associated with the molecular geometry–a huge number *N*^3^ of possible rotamers for *N* rotatable C–C bonds. Along the studied series, *N* increases as 1, 2, 3 and 4. The number of conformers is even higher owing to a various attachment of the hydrogen atoms. One “wrapped” structure of DAVA is visualised in Fig. 2. However, nowadays there are automatic tools such as CREST (Conformer-Rotamer Ensemble Sampling Tool) based on the XTB Semiempirical Extended Tight-Binding for a fast sampling of possible rotamers (Precht et al. 2020).

## Methods

ORCA software, ver. 5.0.4 (Neese et al. 2012, 2020, 2022) has been used for ab initio and DFT calculations of molecular properties and electronic structure of amino acids under study. Actually, the Density Functional Theory with B3LYP hybrid functional was applied (method M1). Alternatively, the HF-MP2 (Hartree–Fock + 2nd-order Many-Body Perturbative Theory with Moller–Plesset partitioning) calculations were exploited (method M2). The basis set def2-TZVPD (triple-zeta-valence with polarisation and diffuse functions) has been applied with the contraction scheme of primitive Gaussians C-, N-, O-{621111/4111/111/1} and H-{311/11} where s-, p-, d- and f-shells are separated by a slash (Weigend and Ahlrichs 2005). The solvent effect has been simulated by Conductor-like Polarizable Continuum Model (CPCM) (Takano and Houk 2005) where the relative permittivity of water was *ε*_r_  = 80.4. The complete vibrational analysis has been performed after the full geometry optimization where no imaginary frequencies were detected.

As a first step, the molecular structures of the amino acids were retrieved from PubChem database; they were used as a starting point for the full geometry optimization until the gradient criteria indicated the global energy minimum. In the case of zwitterionic forms, the crystallographic CCDC data served as an input for the geometry optimization. Then the molecular properties were evaluated: the energies of the HOMO (the highest occupied molecular orbital) and LUMO (the lowest unoccupied molecular orbital), the permanent dipole moment *p*, the quadrupole moment *Q* and the dipole polarizability *α*. The complete vibrational analysis (indicating no imaginary frequencies) facilitated evaluating the electronic, vibration, rotation and translations partition functions allowing to evaluate the zero-point vibration energy, inner energy *U*, enthalpy *H*, entropy *S* and Gibbs energy *G*. Absolute redox potential is calculated using the reaction Gibbs energy on oxidation and/or reduction *E*_abs_^ø^(L^0^/L^*q*^) [V] = – ∆_r_*G*^ø^[J mol^−1^]/*zF*, for *F* = 96,485 C mol^−1^ and *z* = 1.

The positively and/or negatively charged open-shell systems in the unrestricted variant (UKS or UHF) at the optimised geometries of the neutral species and molecular ions allow evaluating the properties which characterise the redox processes forming ionised forms: (i) adiabatic ionisation energy *E*_i_ = *E*^+^–*E*^0^ (for electron withdrawal); (ii) electron affinity *E*_eg_ = *E*^–^–*E*^0^ (for electron attachment); (iii) the chemical potential expressed by the molecular electronegativity $- \mu = \chi = (E_{{\text{i}}} {-}E_{{{\text{eg}}}} ) \, /{2}$ (electronic gradient, a driving force for electron transfer); (iv) chemical hardness $\eta = (E_{{\text{i}}} + E_{{{\text{eg}}}} ) \, /{2}$ (electronic force constant, resistivity against change of electron density); (v) the electrophilicity index *ω* = *χ*^2^/2*η* (electrophilic power), (Sen 1993; Pearson 1977; Parr 1999). The present methodology follows detailed studies on solvation of the glycine molecule, its molecular cation and molecular anion using B3LYP as well as HF-MP2 methods (Boča et al. 2023).

More advanced quantum chemical methods are at the disposal, as implemented in ORCA package. However, the target quantity–the redox potential requires the reaction Gibbs energy that is evaluated after a complete vibrational analysis preceded by the full geometry optimization. DLPNO-CCSD(T) method (Domain-based Local Pair Natural Orbitals–Coupled Cluster Singlets + Doublets + Triplets) is slow when the geometry optimization is activated: for numerical evaluation of gradient in each step of the geometry optimization (dozens to hundreds steps), the energies for a huge number of displacements need to be calculated (for instance 114 displacements for DAVA). Then the time requirement is ca 10^5^ times higher than for a single point calculation that is comparable to more approximate methods like B3LYP (Liakos and Neese 2015; Sandler et al. 2021; Mallick et al. 2020; Garcia-Rates et al. 2021; Paiva et al. 2020). DLPNO-CCSD(T) calculations (method M3) were done in the aug-cc-pVTZ basis set for O-, N-, C-19s6p3d2f contracted to 5s4p3d2f pattern {88111/3111/111/11}, and H-6s3p2d → 4s3p2d{3111/111/11}; auxiliary basis set was aug-cc-pVTZ/C for O-, N-, C- 9s7p6d4f2g {111111111/1111111/111111/1111/11} and H-5s4p3d2f {11111/1111/111/11} (Kendall et al. 1992; Weigend et al. 2002). Augmented basis set is essential for a reliable description of molecular anions.

## Results and discussion

As a result of the geometry optimization, the calculated energies of amino acids in water are listed in Table S1. They include 24 molecules and/or molecular ions calculated by B3LYP/def2-TZVPD method, 12 entries by MP2/def2-TZVPD method and 24 ones by DLPNO-CCSD(T)/aug-cc-pVTZ. The optimised geometries by B3LYP method are drawn in Tables S2 and S3.

Based upon the B3LYP total electronic energy, glycine and β-alanine are more stable in their zwitterionic forms relative to their canonical counterparts (in water): ∆*E* = *E*(Z^0^)–*E*(A^0^) = − 3.9 and − 1.1 kcal mol^−1^, respectively. For GABA and DAVA, on the contrary, a more stable are the canonical amino acid forms: ∆*E* = 0.2 and 0.4 kcal mol^−1^. This finding cannot be overemphasised since we are comparing only two of a huge number of isomers. The MP2/def2-TZVPD calculation gave ∆*E* = − 4.6 and − 2.0 kcal mol^−1^ for **5** and **6**; ∆*E* =− 2.9, 0.1, 1.3, and 1.7 kcal mol^−1^ for **7**–**10** calculated by DLPNO-CCSD(T)/aug-cc-pVTZ.

The calculated total energies of the neutral molecule and its cations and anions allow determining the adiabatic ionisation energy *E*_i_ and adiabatic electron affinity *E*_eg_. Notice, these quantities have little to do with the energies of HOMO and LUMO for the neutral molecule (the Koopmans’ theorem could be valid for the vertical ionisation process). The quantities referring to the adiabatic oxidation and/or reduction processes are listed in Table 1. A comparison of data calculated for the canonical forms brings the following findings. (i) The highest ionisation energy possesses the glycine molecule (147 kcal mol^−1^ for 1A), whereas three remaining molecules have a bit lower values (139, 137 and 136 kcal mol^−1^ for 2A –4A). (ii) The MP2 values of ionisation energies are a bit higher relative to B3LYP data (151 and 146 kcal mol^−1^ for 5A and 6A). (iii) All values of electron affinities are negative (in water as a solvent). (iv) High ionisation energy manifests itself in high molecular electronegativity (*χ* = 86 kcal mol^−1^ is highest for glycine). (v) More negative electron affinity causes the drop of the chemical hardness (*η* = 54–55 kcal mol^−1^) showing that 2A through 4A molecules are “softer” than glycine. (vi) The electrophilicity index reflects the ability of the species to undergo reduction; it is lowest for glycine (*ω* = 61 kcal mol^−1^) which matches the lowest reduction potential along the series (*E*_r_^ø^ = 1.21 V).

**Table 1:** Molecular descriptors calculated by DFT-B3LYP (1–4), HF-MP2 (5, 6) and DLPNO-CCSD(T) (7–10) methods using adiabatic ionisation/affinity processes in water^a^

def2-TZVPD		Adiabatic redox properties							Properties of neutral molecules									
No-method	Molecule	*E*_i_	*E*_eg_	*χ*	*η*	*ω*	*E*_ox_^ø^	*E*_red_^ø^	*p*	*Q*	*α*	*S*	*V*	*E*_zpe_	*S*^ø^*·T*^ø^	HOMO	LUMO	*ν*_0_
Canonical amino acid forms, A^1^																		
1A-M1	Glycine	147	− 25.1	86	61	61	− 6.29	1.21	1.754	− 22.7	56.8	401	623	49.6	22.1	− 163	− 3.4	75.8
2A-M1	β-alanine	139	− 29.7	84	55	64	− 5.98	1.39	1.930	− 26.6	73.9	477	763	67.2	24.7	− 157	− 3.7	8.7
3A-M1	GABA	137	− 29.1	83	54	64	− 5.92	1.38	1.698	− 33.9	90.9	553	903	85.1	26.3	− 155	− 3.0	36.6
4A-M1	DAVA	136	− 28.9	82	54	62	− 5.83	1.36	1.821	− 36.9	107.8	629	1040	102.7	28.6	− 154	− 2.9	14.6
5A-M2	Glycine	151	− 25.0	88	63	61	− 6.48	1.20	1.780	− 22.8	55.4	400	622	50.0	22.0	− 260	38	77.5
6A-M2	β-alanine	146	− 24.6	85	61	60	− 6.27	1.17	1.850	− 26.8	71.8	476	761	67.9	24.3	− 251	38	36.3
7A-M3 ^b^	Glycine	149	− 27.3	88	61	63	–	–	1.891	− 22.7		400	662	–	–	− 260	20.4	–
8A-M3 ^b^	β-alanine	144	− 26.7	85	59	62	–	–	2.124	− 26.6	–	476	761	–	–	− 251	20.3	–
9A-M3 ^c^	GABA	143	− 26.1	84	58	62	–	–	1.770	− 33.9	–	553	903	–	–	− 247	20.6	–
10A-M3 ^c^	DAVA	141	− 25.9	83	57	60	–	–	1.981	− 36.8	–	629	1040	–	–	− 246	20.5	–
Zwitterionic forms, Z																		
1Z-M1	Glycine	150	− 18.9	84	66	53	− 6.45	1.05	13.88	− 21.2	58.8	393	628	50.7	21.8	− 159	− 0.03	74.8
2Z-M1	β-alanine	139	− 18.8	79	60	52	− 6.00	1.11	21.69	− 24.6	76.6	479	766	69.0	24.1	− 148	− 1.6	34.3
3Z-M1	GABA	135	− 11.9	73	62	43	− 5.82	0.52	27.36	− 28.5	93.8	554	905	86.8	26.2	− 144	− 1.1	42.8
4Z-M1	DAVA	133	− 11.4	72	61	42	− 5.74	0.52	33.90	− 32.0	110.8	627	1040	104.6	28.1	− 143	− 1.0	50.2
5Z-M2	Glycine	163	− 14.1	88	74	52	− 7.02	0.86	13.86	− 21.3	57.8	392	625	51.1	21.8	− 257	39	72.1
6Z-M2	β-alanine	155	− 14.6	85	70	51	− 6.69	0.89	21.60	− 24.7	74.8	477	763	69.7	23.9	− 250	40	55.6
7Z-M3^b^	Glycine	156	− 18.1	87	69	55	–	–	14.37	− 21.1	–	392	625	–	–	− 261	20.0	–
8Z-M3^b^	β-alanine	148	− 18.6	83	65	53	–	–	22.02	− 24.5	–	477	763	–	–	− 250	18.9	–
9Z-M3^c^	GABA	144	− 19.9	82	62	54	–	–	27.82	− 28.4	–	554	905	–	–	− 247	18.7	–
10Z-M3^c^	DAVA	142	− 20.1	81	61	54	–	–	34.35	− 32.0	–	627	1040	–	–	− 244	18.6	–
Nickname		I	A	X	H	O	Eo	Er	p	Q	al	S	V	Z	ST	Ho	Lu	n

^a^All energy quantities in units of kcal mol^−1^; conversion: 1 hartree = 627.503 kcal mol^−1^; 1 eV = 23.0609 kcal mol^−1^; 1 kcal mol^−1^ = 4.184 kJ mol^−1^. Standard temperature *T*^ø^ = 298.15 K. Redox properties: ionisation energy *E*_i_ = *E*^+^−*E*^0^, electron affinity *E*_eg_ = *E*^−^−*E*^0^; molecular electronegativity $\chi = (E_{{\text{i}}} {-}E_{{{\text{eg}}}} ) \, /{2}$, chemical hardness $\eta = (E_{{\text{i}}} + E_{{{\text{eg}}}} ) \, /{2}$, electrophilicity index $\omega = \chi^{2} /2\eta$, absolute oxidation potential *E*_ox_^ø^ = $- \Delta_{{{\text{ox}}}} E({\text{L}}^{0} /{\text{L}}^{ + } )/F$ in V, absolute reduction potential *E*_red_^ø^ = $- \Delta_{{{\text{red}}}} E({\text{L}}^{0} /{\text{L}}^{ - } )/F$ in V, Faraday constant 96,485 A s mol^−1^. Dipole moment *p*/D; isotropic quadrupole moment *Q*/e*a*_0_^2^, isotropic dipole polarizability *α*/*a*_0_^3^, solvated surface area *S*/*a*_0_^2^, solvated volume *V*/*a*_0_^3^, *debye*, *D* = 3.336 × 10^–30^ Ams; *angstrom*, Å = 10^–10^ m; *bohr*, *a*_0_ = 5.292 × 10^−11^ m; zero-point energy *E*_zpe_, lowest vibrational (harmonic) frequency *ν*_0_/cm^−1^, total entropic term *S·T*^*ø*^

^b^Calculated by DLPNO-CCSD(T) method using aug-cc-pVTZ and aug-cc-pVTZ/C basis set in the fixed geometry optimised by MP2/def2-TZVPD

^c^Calculated by DLPNO-CCSD(T) method using aug-cc-pVTZ and aug-cc-pVTZ/C basis set in the fixed geometry optimised by B3LYP/def2-TZVPD

The molecular properties related to the electroneutral molecules vary systematically with the bulkiness of the species: the isotropic value of the quadrupole moment *Q* = − 23, − 27, − 34 and − 37 *ea*_0_^2^, the isotropic value of the dipole polarizability *α* = 57, 74, 91 and 108 *a*_0_^3^, the solvated surface *S* and solvated volume *V*, the zero-point vibrational energy *E*_zpe_ = 49.6, 67.2, 85.1 and 102.7 kcal mol^−1^, and the total entropic terms *S·T*^ø^ = 22, 25, 27 and 29 kcal mol^−1^. The dipole moment *p* = 1.75, 1.93, 1.70 and 1.82 D does not vary systematically because it depends upon the delicate spatial separation of the barycenters of opposite charges.

A comparison of data calculated by MP2 method with those obtained by B3LYP shows some differences which, however, are not principal and do not alter the conclusions: the calculated bulk properties (*Q*, *α*, *S*, *V*, *E*_zpe_, *S·T*^ø^) are almost the same. The main difference lies in the energies of one-electron orbitals HOMO and LUMO which have no physical impact (e.g. for glycine: − 163 *vs* − 260 for HOMO, and − 3 *vs* + 38 kcal mol^−1^ for LUMO); these, however, have no impact to observables, such as ionisation energies and electron affinities. The adiabatic ionisation energy and the electron affinity are little affected when comparing B3LYP and MP2 data (for glycine: *E*_i_ = 147 *vs* 151, *E*_eg_ = − 25.1 *vs* − 25.0 kcal mol^−1^). The derived electronic properties, such as molecular electronegativity, chemical hardness and electrophilicity index, are very similar (for glycine: *χ* = 86 *vs* 88, *η* = 61 *vs* 63, *ω* = 61 *vs* 61 kcal mol^−1^); the same hold true for the absolute reduction potential (*E*_r_^ø^ = 1.21 *vs* 1.20 V).

The molecular descriptors calculated by DLPNO-CCSD(T) method are restricted to single-point energy calculations (including molecular ions), i.e. the dipole moment, quadrupole moment, ionisation energy and electron affinity along with derived electronic parameters, such as the molecular electronegativity, chemical hardness and the electrophilicity index. These data are almost the same as obtained by MP2 (for glycine: *E*_i_ = 151 *vs* 149, *E*_eg_ = − 25.0 *vs* − 27.3 kcal mol^−1^; for β-alanine: *E*_i_ = 146 vs 144, *E*_eg_ = − 24.6 *vs* − 26.7 kcal mol^−1^). Then the derived electronic properties are also analogous (for glycine: *χ* = 88 *vs* 88, *η* = 63 *vs* 61, *ω* = 61 *vs* 63; for β-alanine: *χ* = 85 *vs* 85, *η* = 61 *vs* 59, *ω* = 60 *vs* 62 kcal mol^−1^).

When comparing all four members calculated by DLPNO-CCSD(T) method (**7** through **10**), some systematic trends are recognised (all energy data in kcal mol^−1^): (i) the ionisation energy decreases as *E*_i_ = 149, 144, 143, 141; (ii) the electron affinity varies as *E*_eg_ = − 27.3, − 26.7, − 26.1 − 25.9; (iii) the molecular electronegativity decreases as *χ* = 88, 85, 84, 83; (iv) the chemical hardness decreases as *η* = 61, 59, 58, 57; (v) the electrophilicity index decreases as *ω* = 63, 62, 62, 60. The quadrupole moment varies as *Q* = − 23, − 27, − 34 and − 37 *ea*_0_^2^; however, the dipole moment varies non-systematically as *p* = 1.89, 2.12, 1.77 and 1.98 D owing to specific separations of barycenters of the positive–negative charges.

The same set of molecular properties has been calculated for the zwitterionic (Z) forms of the amino acids, after full geometry optimization and complete vibrational analysis for the neutral and ionised species using B3LYP/def2-TZVPD and MP2/def2-TZVPD methods (Table 2). One can see: (i) the values of the adiabatic electron affinities are much lower (in absolute values) relative to canonical forms; (ii) consequently the electrophilicity index is much lower; (iii) the reduction potential drops substantially; (iv) according to the chemical hardness, the zwitterionic forms are more hard.

**Table 2:** Comparison of calculated and experimental data^a,b^

Method^c^		UPS (vac)	Canonical form (w)			Zwitterion (w)		
		*E*_i_(v)	*E*_i_(a)	*p*/D	*α*/a_0_^3^	*E*_i_(a)	*p*/D	*α*/a_0_^3^
Glycine								
Experiment [c], [f], [b], [d]		v: 230 [10.0] a: 203 [8.8]					11.9	44.2
B3LYP/def2-TZVPD, in water [this work]	M1	v: 224 (vac)	147	1.75	56.8	150	13.9	58.8
DLPNO-CCSD(T)/aug-cc-pVTZ, in water [t.w.]	M3	v: 235 (vac)	149	1.89		156	14.4	
MP2/def2-TZVPD, in water [this work]	M2	v: 237 (vac)	151	1.78	55.4	163	13.9	57.8
MP2, CCSD(T), in vacuo [a]	M2 M3			1.29 (vac) 1.28 (vac)	43.1 (vac) 43.0 (vac)			
B97-1/cc-pVDZ, in vacuo [a]		v: 220 (vac)		1.32 (vac)	43.6 (vac)			47.1 (vac)
*β*-alanine								
Experiment [f], [e]		v: 223 [9.7]					19.4	
B3LYP/def2-TZVPD, in water [this work]	M1	v: 216 (vac)	139	1.93	73.9	139	21.7	76.6
DLPNO-CCSD(T)/aug-cc-pVTZ, in water [t.w.]	M3	v: 227 (vac)	144	2.12		148	22.0	
MP2/def2-TZVPD, in water [this work]	M2	v: 229 (vac)	146	1.85	71.8	155	21.6	74.8
GABA								
Experiment [f]		v: 221 [9.6]						
B3LYP/def2-TZVPD, in water [this work]	M1	v: 210 (vac)	137	1.70	90.9	135	27.4	93.8
DLPNO-CCSD(T)/aug-cc-pVTZ, in water [t.w.]	M3	v: 220 (vac)	143	1.77		144	27.8	
DAVA								
Experiment [f]		v: 217 [9.4]						
B3LYP/def2-TZVPD, in water [this work]	M1	v: 207 (vac)	136	1.82	107.8	133	33.9	110.8
DLPNO-CCSD(T)/aug-cc-pVTZ, in water [t.w.]	M3	v: 221 (vac)	141	1.98		141	34.3	

^a^Energies in kcal mol^−1^ [eV]; 1 eV = 23.0609 kcal mol^−1^. Ionisation energies *E*_i_: *a*–adiabatic, *v*–vertical; vertical ionisation energy always is higher than the adiabatic ionisation energy. (w, aq)–in water; (vac, g)–in vacuo; *p–*dipole moment in *debye*, *D* = 3.336 × 10^–30^ Ams; units for polarizability *α*_*ij*_ = d*p*_*i*_/d*E*_j_: *a*_0_^3^ = 0.14818 Å^3^, *α*[Å^3^] = 10^–24^ × *α*[cm^3^], *α*[cm^3^] = * α*[SI = C^2^ m^2^ J^−1^] × 106/4πε_0_, *α*[a03] = 1.648777 × 10–41 α[C^2^ m^2^ J^−1^], *α*[SI] = * α*(a03) × 6.0651 × 1040 C m^2^ V^−1^]

^b^References: [a] Calculations (Millefiori et al. 2008); [b] Kerr effect (Khanarian and Moore 1980); [c] UPS (Campbell et al. 1992); [d] Dielectric permittivity (Sato et al. 2005); [e] Dielectric permittivity (Aaron and Grant 1967); [f] UPS (Cannington and Ham 1983)

^c^B3LYP calculations in the fully optimised geometry of the neutral molecule and its cation; DLNPO-CCSD(T) calculations done in the fixed geometry resulting from the optimization by B3LYP (for GABA and DAVA) or MP2 method (for glycine and *β*-alanine)

DLPNO-CCSD(T) method within the series 7Z through 10Z gave the following systematic trends: (i) the ionisation energy decreases as *E*_i_ = 156, 148, 144, 142; (ii) the electron affinity varies as *E*_eg_ = − 18.1, − 18.6, − 19.9, − 20.1; (iii) the molecular electronegativity decreases as *χ* = 87, 83, 82, 81; (iv) the chemical hardness decreases as *η* = 69, 65, 62, 61; (v) the electrophilicity index varies as *ω* = 55, 53, 54, 54 kcal mol^−1^; (vi) the dipole moment increases as *p* = 14, 22, 28 and 34 D; and (vii) the quadrupole moment varies as *Q* = − 21, − 24, − 28, − 32 *ea*_0_^2^.

**Fig. 3:**
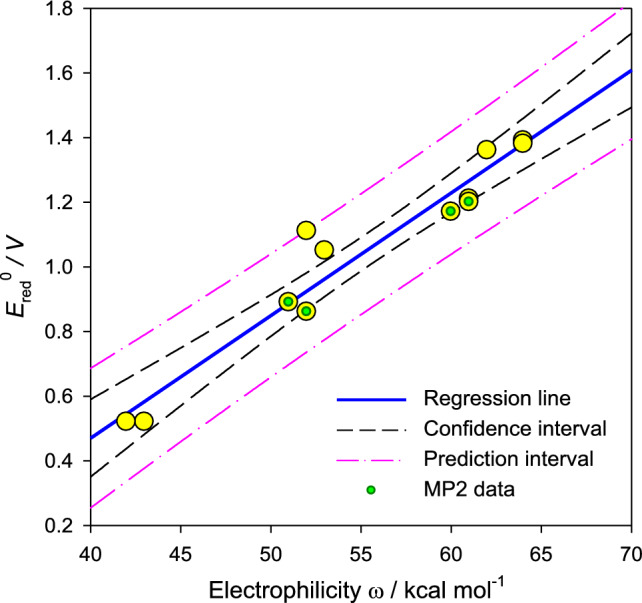
A correlation of the absolute reduction potential with the electrophilicity index. Yellow circles–full data set, solid points–MP2 data. Regression line: *E*_red_^ø^ [V] = − 1.05 [V] + 0.038 *ω* [kcal mol^−1^]; regression coefficient *r*^2^ = 0.94

The absolute reduction potential correlates with the electrophilicity index *ω*: such a correlation is displayed in Fig. 3. Note that *ω* is a property derived from the electronic energy of L^0^, L^+^ and L^−^ species whereas the reduction potential is a thermodynamic quantity derived from the reaction Gibbs energy between L^−^ and L^0^.

A comparison of the calculated data in solution with experimental ones is rather problematic since the available data refer mostly to the proteinogenic *α*-amino acids *in vacuo*. The same holds for the ab initio calculations usually done without a solvent effect. A collection of calculated and experimental data is presented in Table 2. This table contains data about ionisation energies as obtained by the Ultra-Violet Photoelectron Spectroscopy (UPS) in the gaseous phase. The vertical ionisation energies were also calculated by various methods *in vacuo* for testing their reliability (energy of the neutral species refers to the optimised geometry, for molecular cation to the frozen geometry of neutral molecule). This process is assigned as an ionisation of the nitrogen lone pair (abbr. n).

The vertical ionisation energy in the gaseous phase of glycine, *E*_i_(v) = 230 kcal mol^−1^, is well reproduced by all methods: M1–224, M2–237 and M3–235 kcal mol^−1^. For β-alanine, *E*_i_(v) = 223 kcal mol^−1^ is compared by M1–216, M2–229 and M3–227 kcal mol^−1^; for GABA, *E*_i_(v) = 221 kcal mol^−1^ is reproduced by M1–210 and M3–220 kcal mol^−1^; for DAVA, *E*_i_(v) = 217 kcal mol^−1^ is compared by M1–207 and M3–221 kcal mol^−1^. It can be concluded that B3LYP method offers vertical ionisation energies that are a bit lower (− 5%) relative to the gas-phase UPS data; the DLPNO-CCSD(T) method, on the contrary a bit higher (+ 2%). Their trends are correctly reproduced by all applied methods.

The dipole moment measured in an aqueous solution with dominating zwitterionic form does not reflect a single molecular unit because of existence various rotamers in a complex equilibrium. The agreement with M1, M2 and M3 methods in not perfect, however, it is acceptable.

**Fig. 4:**
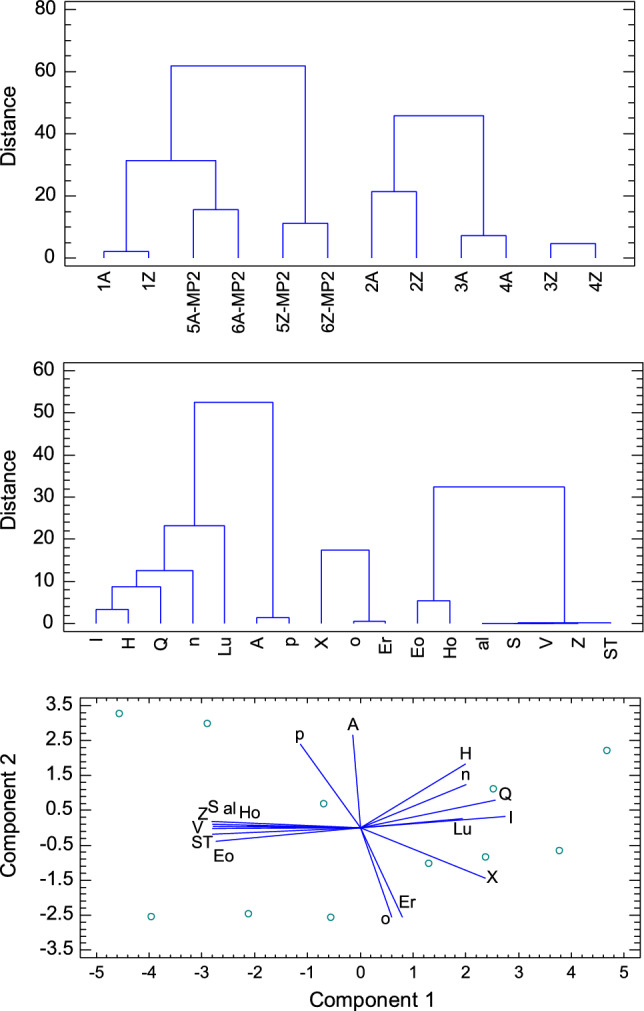
Results of the Cluster Analysis (top and centre) and the Principal Component Analysis (bottom) using dataset from Table 2

The calculated molecular descriptors served as a worksheet for advanced statistical methods (Statgraphics 1982). The Cluster Analysis (CA) is a kind of the classification statistical methods that forms similar objects or observables into groups according to their “distance”. A squared Euclidean distance metric has been applied along with the Wards method. Results are displayed in Fig. 4 for objects (molecules) and observables (molecular properties). It is seen that 12 molecules can be classified into three groups. The bulk properties form own group: polarizability (a), solvated surface (S) and volume (V), zero-point energy (Z) and total entropic term (ST); high degree of similarity (low distance) show electrophilicity index (o) and the reduction potential (Er).

The Principal Component Analysis (PCA) belongs to the multivariate statistical methods making “principal components” formed of linear combination of variables that contain the greatest variance. PCA biplot is drawn in Fig. 4 (bottom) showing which molecular properties correlate/anti-correlate/non-correlate. It matches the classification by CA: variables located in adjacent rays mutually correlate, e.g. {O, Er}, {(al, Z, S, V, ST), (Eo, Ho)}.

The calculated pair correlation coefficients are listed in Table 3; high values *ρ*(A, B) > 0.95 point to significant correlations. Amongst them, the absolute oxidation potential (calculated from the reaction Gibbs energy) correlates with the adiabatic ionisation energy (calculated from the electronic energies only). Analogously, the absolute reduction potential correlates with the adiabatic electron affinity (this has nothing to do with LUMO) and also with the electrophilicity index (Fig. 2). A matrix of scatter points referring to the molecular descriptors of amino acids is deposited in Supplementary Information.

**Table 3:** Pair correlation coefficients amongst molecular descriptors

*ρ*(A, B)	I	A	X	H	O	P	al	Er	Eo
I		− 0.19	0.70	0.52	0.27	− 0.38	− 0.91	0.33	− **1.00**
A	− 0.19		− 0.83	0.74	− **1.00**	**0.96**	0.21	− **0.96**	0.17
X	0.70	− 0.83		− 0.25	0.87	− 0.91	− 0.67	0.89	− 0.69
H	0.52	0.74	− 0.25		− 0.69	0.58	− 0.43	− 0.62	− 0.53
O	0.27	− 1.00	0.87	− 0.69		− **0.98**	− 0.28	**0.98**	− 0.25
p	− 0.38	0.96	− 0.91	0.58	− 0.98		0.36	− 0.93	0.35
al	− 0.91	0.21	− 0.67	− 0.43	-0.28	0.36		− 0.35	0.92
Er	0.33	− 0.96	0.89	− 0.62	0.98	− 0.93	− 0.35		− 0.31
Eo	− 1.00	0.17	− 0.69	− 0.53	− 0.25	0.35	0.92	− 0.31	

*I *adiabatic ionisation energy, *A* electron affinity, *Xi* molecular electronegativity, *H* chemical hardness, *om* electrophilicity index, *p* dipole moment, *al* polarizability, *Er* reduction potential, *Eo* oxidation potential

The calculated vibrational frequencies are compared in Table 4; they form the vibrational spectra displayed in Figure S2. A comparison of scaled MP2 with experimental data taken *in vacuo* is possible; an agreement is satisfactory. This data can be compared with those calculated by other versions of DFT (Barone et al. 2013).

**Table 4:** Calculated (unscaled) harmonic vibrational frequencies [cm^−1^] for glycine in the canonical form **1** and zwitterionic form **Z** by MP2 and B3LYP methods^a^

Exptl	MP2	MP2	MP2	B3LYP	MP2	B3LYP
In vacuo	**1**, in vacuo	**1**, in vacuo, scaled	**1**, in water	**1**, in water	**Z**, in water	**Z**, in water
n.a	71	68	77	76	72	75
204	216	208	239	230	208	219
250	261	252	262	258	288	283
458	470	453	473	467	503	495
500	518	499	517	506	577	574
615	639	616	629	628	676	669
619	650	627	640	636	876	862
801	833	803	840	820	930	924
883	922	889	922	917	1041	992
907	929	895	942	917	1094	1091
1101	1137	1096	1129	1110	1108	1099
1136	1177	1134	1173	1155	1314	1304
1166	1193	1150	1197	1192	1328	1329
1297	1308	1261	1312	1308	1385	1367
1340	1393	1343	1390	1382	1430	1434
1405	1412	1361	1415	1397	1477	1466
1429	1469	1416	1448	1437	1619	1612
1608	1673	1613	1638	1641	1642	1631
1779	1809	1744	1771	1754	1651	1640
2943	3079	2968	3076	3043	3132	3106
2969	3128	3015	3130	3077	3193	3162
3359	3506	3379	3491	3485	3205	3171
3410	3590	3460	3576	3553	3484	3471
3585	3728	3593	3696	3688	3550	3523

^a^Experimental data include anharmonic frequencies

## Conclusions

In conclusion, three quantum chemical methods were used to calculate the electronic properties of linear amino acids (glycine, β-alanine, GABA and DAVA) in water as a solvent: B3LYP/def2-TZVPD (a hybrid variant of the Density Functional Theory; method M1), HF-MP2/def2-TZVPD (the 2nd-order many-body perturbation theory to the correlation energy; method M2), and DLPNO-CCSD(T)/aug-cc-pVTZ (a variant of the coupled-cluster method with excitations of singlets, doublets and triplets included in the correlation energy; method M3). Each species was considered in the canonical amino acid (A) form as well as in the zwitterionic (Z) form as an electroneutral molecule L^0^, its molecular cation L^+^ and molecular anion L^−^. A total of 60 systems were examined. Full geometry optimization followed by complete vibrational analysis was performed with M1 for all four amino acids, and by M2 for glycine and β-alanine. M3 calculations were performed in geometries optimised by M1 or M2 methods.

The present work has certain advantages compared to the fragmentary quantum chemical studies found in literature.

1. The selected objects (linear *ω*-amino acids) are members of a series along which the carboxyl group is monotonously separated from the terminal amino group. It would be beneficial if this series will continue with branched-chain *α*-amino acids, sulphur containing amino acids, etc., to accumulate extensive data set on the molecular properties of different types of amino acids generated by a consistent methodology.
2. All studies were done in water as a solvent, where experimental data is very lacking.
3. Before the conclusions, the ability to reproduce experimental data in vacuum was tested using the B3LYP method, MP2 method, and the DLPNO-CCSD(T) method, taking into account vertical and adiabatic ionisation energies from UPS spectra, and vibrational spectra.
4. The calculated properties include two classes: (i) energetic and electrical properties of neutral species, such as dipole moment *p* (measure of polarity, or charge separation), quadrupole moment *Q* (measure of eccentricity of the charge cloud), dipole polarizability *α* (the ability of the electron cloud to be distorted in an electric field), solvated surface *S* and solvated volume *V*, zero-point vibrational energy *E*_zpe_, and total entropic term *S*^ø^*·T*^ø^; (ii) properties that characterise the redox processes forming ionised forms, such as adiabatic ionisation energy *E*_i_ (for electron withdrawal), electron affinity *E*_eg_ (for electron attachment), chemical hardness *η* (electronic force constant, resistance to change in electron density), molecular electronegativity *χ* (electronic gradient, driving force of electron transfer), electrophilicity index *ω* (electrophilic power), absolute oxidation *E*_ox_^ø^ and reduction *E*_red_^ø^ potentials (thermodynamic driving forces for oxidation/reduction).
5. According to B3LYP data for the canonical forms of amino acids, the set of bulk properties shows a monotonic increase with the (molar) mass of the molecule: |*Q*| (23–37 *ea*_0_^2^), *α* (57–108 *a*_0_^3^), *S* (401–629 *a*_0_^2^), *V* (623–1040 *a*_0_^3^), *E*_zpe_ (50–103 kcal mol^−1^) and *S*^ø^*·T*^ø^ (22–29 kcal mol^−1^). The dipole moment varies unsystematically because it depends on the spatial separation of the opposite charges, *p* (1.75, 1.93, 1.70 and 1.82 debye). For zwitterionic forms of amino acids, these properties are very similar: |*Q*| (21–32 *ea*_0_^2^), *α* (59–111 *a*_0_^3^), *S* (393–627 *a*_0_^2^), *V* (628–1040 *a*_0_^3^), *E*_zpe_ (52–105 kcal mol^−1^) and *S*^ø^*·T*^ø^ (22–28 kcal mol^−1^). However, the dipole moment increases dramatically: *p* (14–34 *debye*).
6. For canonical forms, the electronic properties are independent of molar mass and they can vary either systematically, such as *E*_i_ (147–136 kcal mol^−1^) and *E*_ox_^ø^ (− 6.3 to − 5.8 V), or less systematically, such as *E*_eg_ (− 25 to − 30 kcal mol^−1^), *E*_red_^ø^ (1.21–1.39 V), *χ* (86–82 kcal mol^−1^), *η* (61–54 kcal mol^−1^) and *ω* (61–64 kcal mol^−1^). For zwitterionic forms: *E*_i_ (150–135 kcal mol^−1^) and *E*_ox_^ø^ (− 6.4 to − 5.7 V), *E*_eg_ (− 19 to − 11 kcal mol^−1^), *E*_red_^ø^ (1.11–0.52 V), *χ* (84–72 kcal mol^−1^), *η* (66–61 kcal mol^−1^) and *ω* (53–42 kcal mol^−1^). There is a significant difference between the first pair (glycine, β-alanine) and the second pair (GABA, DAVA).
7. Calculations confirm that in water as a solvent: (i) glycine zwitterion is the most stable forms by 4–2 kcal mol^−1^ relative to the canonical form, whilst for GABA and DAVA, it is less stable by 0.5–2 kcal mol^−1^; (ii) the dipole moment is extremely high, 14–34 *debye*; (iii) the absolute reduction potential is moderate, 1.5 V for glycine and β-alanine (stronger oxidising agents), and 0.5 V for GABA and DAVA (weaker oxidising agents); (iv) the absolute reduction potential correlates with the electrophilicity index.
8. With a representative set of objects and their properties, advanced statistical methods (Cluster analysis, Principal component analysis) were used to unhide latent correlations between molecular properties.

Based on similarity of the results obtained with B3LYP and the more exact DLPNO-CCSD(T), it can be concluded that B3LYP cannot currently be discriminated as a method of low confidence, at least for a set of linear amino acids. Its advantage lies in rapid geometry optimization and fast vibrational analysis allowing to evaluate the absolute redox potentials on the basis of the reaction Gibbs energies.

## Supplementary Information

Below is the link to the electronic supplementary material.

Supplementary file1 (PDF 697 KB)

## Data Availability

All computational protocols are available from the corresponding author on request.
