# Supplementary material for: Molecular properties of linear amino acids in water
Source: Amino Acids. 2024 Feb 1;56:5. doi: 10.1007/s00726-023-03365-3 (PMC10834582; doi:10.1007/s00726-023-03365-3)
Supplement: Supplementary file 1 [file 726_2023_3365_MOESM1_ESM.pdf]

Molecular properties of linear amino acids in water

Roman Boča · Richard Imrich · Juraj Štofko · Beáta Vranovičová · Cyril Rajnák

University of SS Cyril and Methodius, 917 01 Trnava, Slovakia

Supplementary information

Table S1 Energies in optimum geometries of amino acids in water by DFT-B3LYP, MP2 and DLPNO-CCSD(T) methods. [a]

Molecule	Canonical form			Zwitterionic form			ΔE
	A^+	A^0	A^-	Z^+	Z^0	Z^-	ΔG^o
**1**, glycine, B3LYP/def2-TZVPD							
Electronic energy E^q	-178332.16	-178479.28	-178504.36	-178333.54	**-178483.14**	-178502.06	-3.9
Gibbs energy $G^{o,q}$	-178302.51	-178447.67	-178475.68	-178301.55	-178450.27	-178474.53	-2.6
**2**, β-alanine, B3LYP/def2-TZVPD							
Electronic energy E^q	-203000.85	-203139.68	-203169.42	-203001.84	**-203140.83**	-203160.62	-1.1
Gibbs energy $G^{o,q}$	-202954.43	-203092.23	-203124.20	-202952.84	-203091.17	-203116.66	+1.1
**3**, GABA, B3LYP/def2-TZVPD							
Electronic energy E^q	-227663.42	**-227800.89**	-227830.01	-227665.70	-227800.72	-227812.63	+0.2
Gibbs energy $G^{o,q}$	-227599.77	-227736.26	-227768.16	-227600.27	-227734.44	-227746.40	+1.8
**4**, DAVA, B3LYP/def2-TZVPD							
Electronic energy E^q	-252325.10	**-252461.03**	-252489.97	-252327.37	-252460.67	-252472.03	+0.4
Gibbs energy $G^{o,q}$	-252245.66	-252380.20	-252411.65	-252245.23	-252377.68	-252389.67	+2.5
**5**, glycine, MP2/def2-TZVPD							
Electronic energy E^q	-178101.13	-178251.76	-178276.75	-178093.74	**-178256.40**	-178270.50	-4.6
Gibbs energy $G^{o,q}$	-178070.33	-178219.67	-178247.40	-178061.06	-178223.11	-178242.91	-3.4
**6**, β-alanine, MP2/def2-TZVPD							
Electronic energy E^q	-202727.11	-202873.02	-202897.58	-202720.08	**-202875.06**	-202889.64	-2.0
Gibbs energy $G^{o,q}$	-202679.81	-202824.43	-202851.46	-202670.19	-202824.51	-202845.11	-0.1
**7**, glycine, DLPNO-CCSD(T)/aug-cc-pVTZ [b]							
Electronic energy E^q	-178150.84	-178299.52	-178326.78	-178146.09	**-178302.40**	-178320.53	-2.9
**8**, β-alanine, DLPNO-CCSD(T)/aug-cc-pVTZ [b]							
Electronic energy E^q	-202793.56	**-202937.38**	-202964.10	-202789.35	-202937.33	-202955.97	+0.1
**9**, GABA, DLPNO-CCSD(T)/aug-cc-pVTZ [c]							
Electronic energy E^q	-227432.97	**-227575.55**	-227601.65	-227430.25	-227574.20	-227594.15	+1.3
**10**, DAVA, DLPNO-CCSD(T)/aug-cc-pVTZ [c]							
Electronic energy E^q	-252071.83	**-252213.01**	-252238.91	-252068.96	-252211.32	-252231.39	+1.7

[a] Energies in kcal mol^{-1}; used conversion: 1 hartree = 627.503 kcal mol^{-1}. Energy of a more stable neutral isomer is **bold** typed. $\Delta E = E(Z^0) - E(A^0)$; $\Delta G = G(Z^0) - G(A^0)$.
[b] Calculated by DLPNO-CCSD(T) method using aug-cc-pVTZ and aug-cc-pVTZ/C basis sets in the fixed geometry optimized by MP2/def2-TZVPD for neutral molecule and molecular ions.
[c] Calculated by DLPNO-CCSD(T) method using aug-cc-pVTZ and aug-cc-pVTZ/C basis sets in the fixed geometry optimized by B3LYP/def2-TZVPD for neutral molecule and molecular ions.

Table S2 Optimized geometries of amino acids (canonical forms) in water by DFT-B3LYP/def2-TZVPD.

Canonical A[1] form	L^+	L^0	L^-
glycine	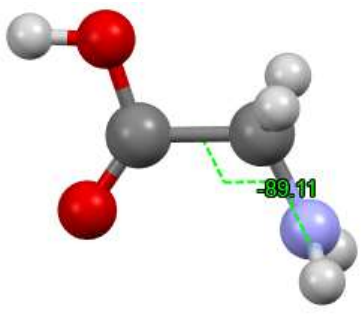 C-C-N-H = -89, 90 deg	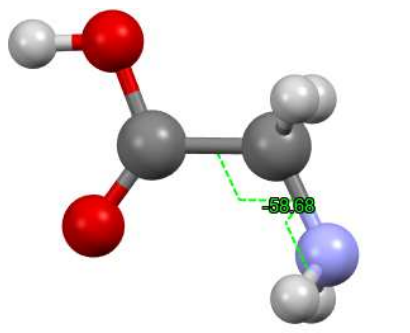 C-C-N-H = -59, 60 deg<br>C…N = 2.52 Å, *p* = 1.754 D	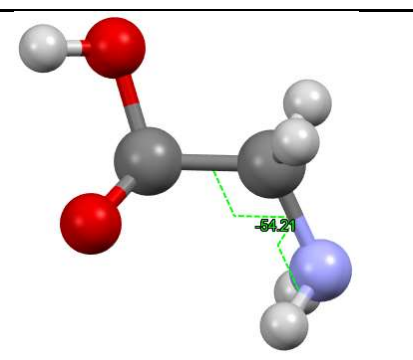 C-C-N-H = -54, 58 deg
β-alanine	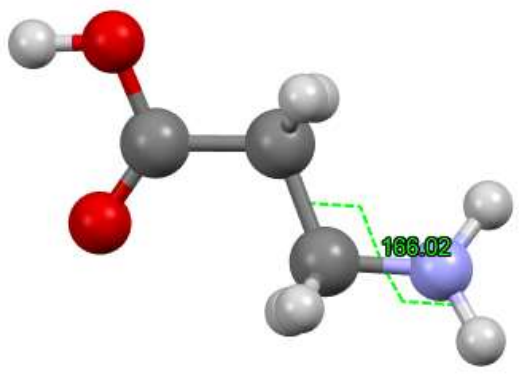 C-C-N-H = -18, 166 deg	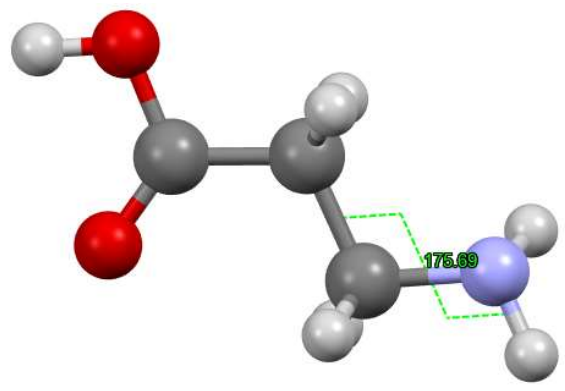 C-C-N-H = -68, 176 deg<br>C…N = 3.81 Å, *p* = 1.930 D	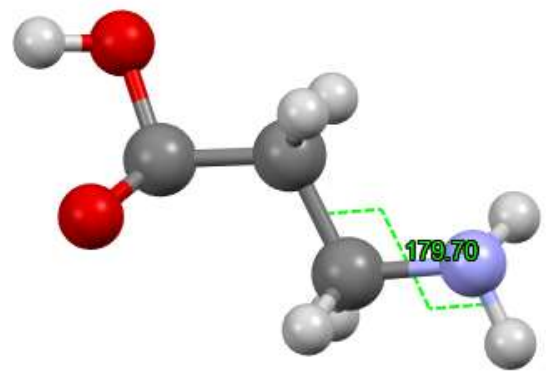 C-C-N-H = -64, 180 deg
GABA	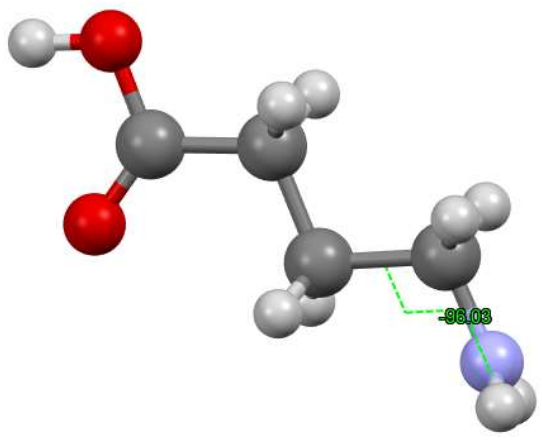 C-C-N-H = -96, 78 deg	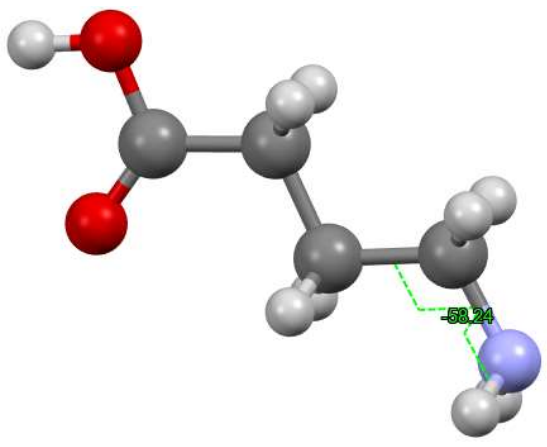 C-C-N-H = -58, 58 deg<br>C…N = 5.07 Å, *p* = 1.698 D	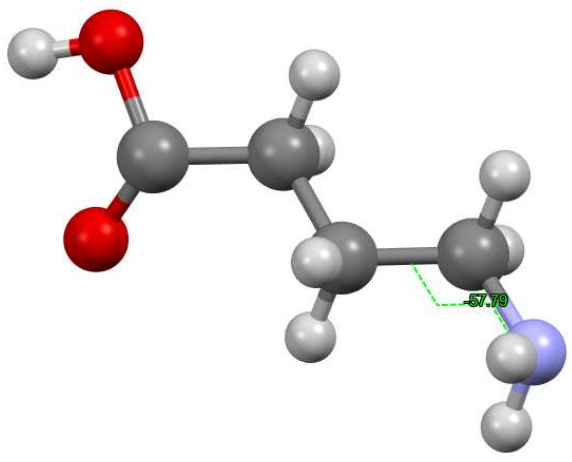 C-C-N-H = -58, 58 deg
DAVA	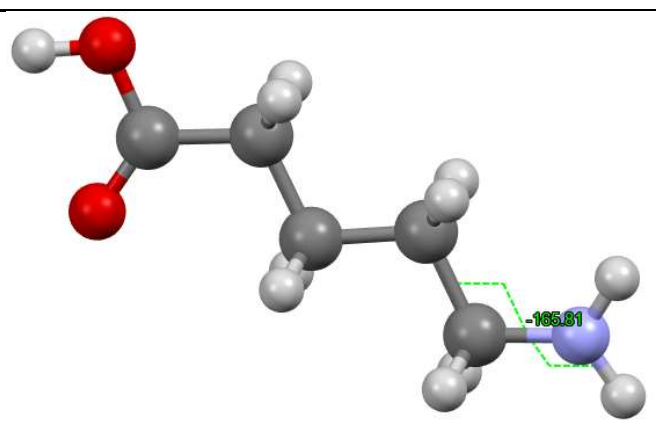 C-C-N-H = -166, 18 deg	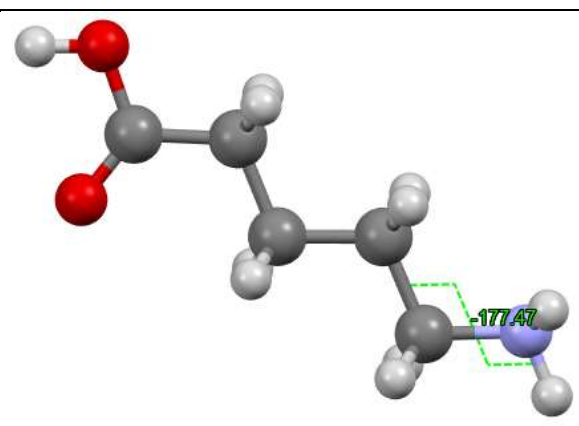 C-C-N-H = -177, 66 deg<br>C…N = 6.39 Å, *p* = 1.821 D	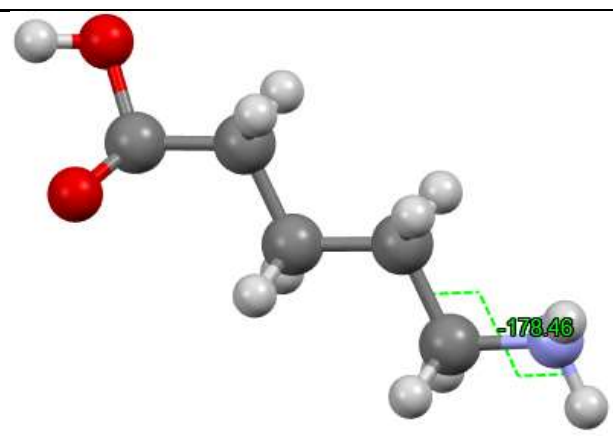 C-C-N-H = -178, 65

C…N is the separation of the carboxylic carbon and amine nitrogen.

Table S3 Optimized geometries of amino acids (zwitterionic forms) in water by DFT-B3LYP/def2-TZVPD.

Zwitterionic forms	L^+	L^0	L^-
glycine	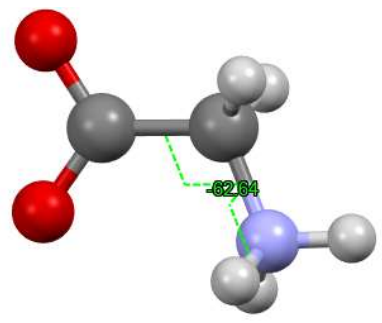<br>C-C-N-H = -62, 59, 178 deg	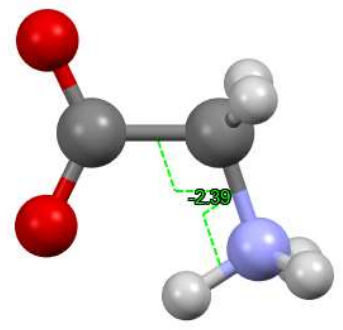<br>C-C-N-H = -121, -2, 116 deg<br>C…N = 2.47 Å, *p* = 13.88 D	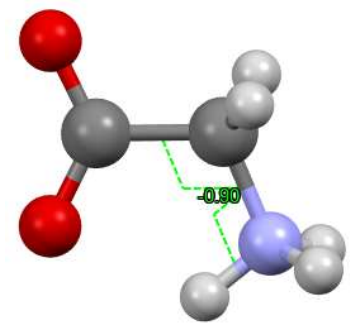<br>C-C-N-H = -118, -1, 120 deg
β-alanine	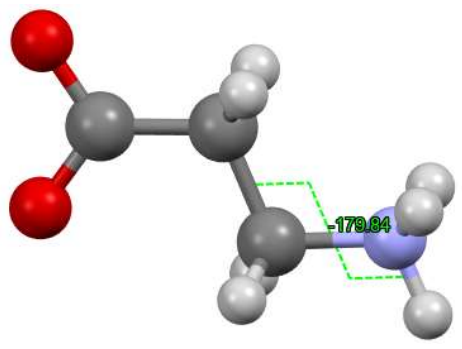<br>C-C-N-H = -180, -60, 60 deg	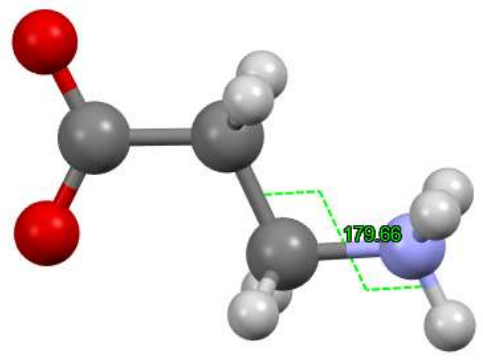<br>C-C-N-H = -60, 60, 180 deg<br>C…N = 3.89 Å, *p* = 21.69 D	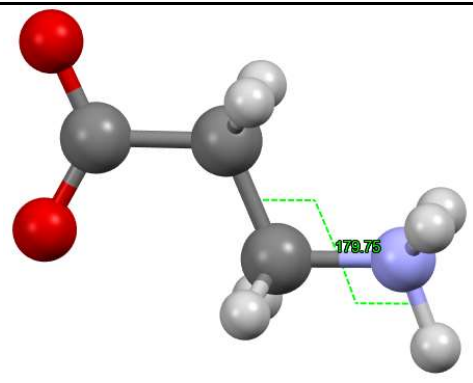<br>C-C-N-H = -60, 60, 180 deg
GABA	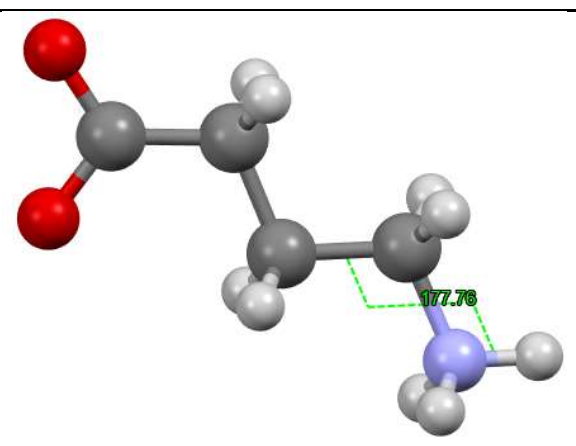<br>C-C-N-H = -62, 58, 178 deg	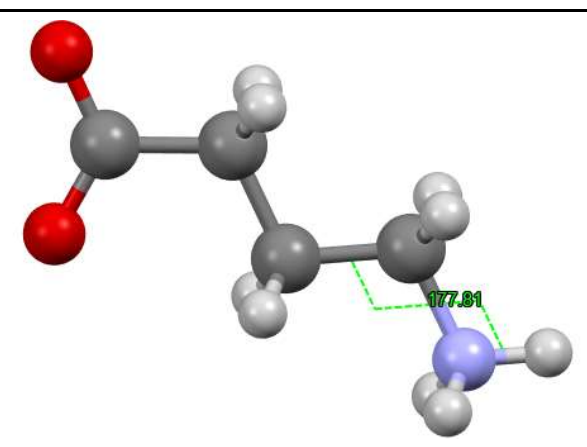<br>C-C-N-H = -62, 58, 178 deg<br>C…N = 5.09 Å, *p* = 27.36 D	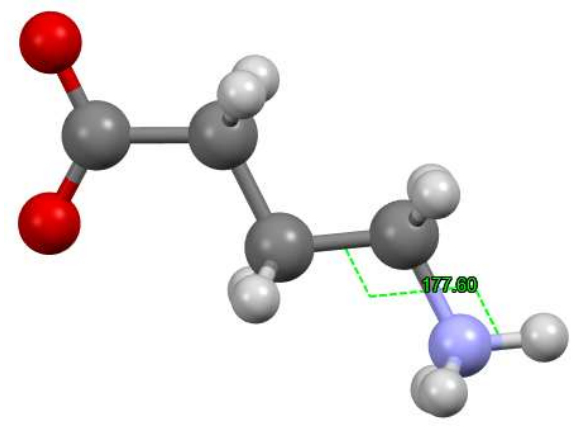<br>C-C-N-H = -62, 58, 178 deg
DAVA	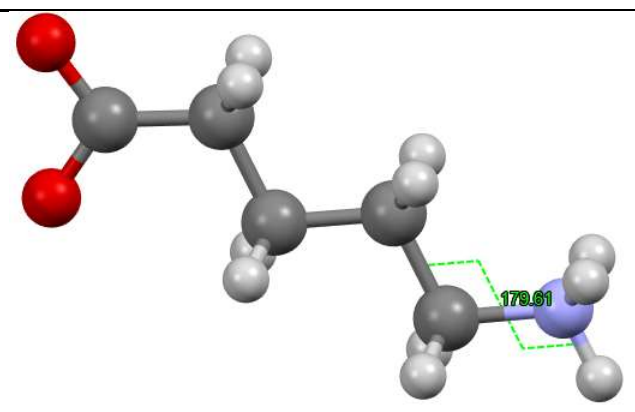<br>C-C-N-H = -60, 60, 180 deg	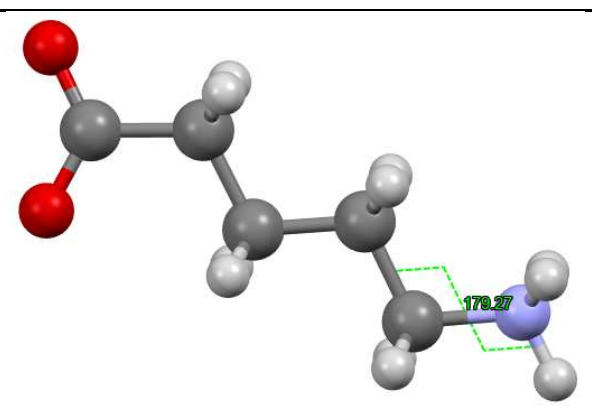<br>C-C-N-H = -61, 59, 180 deg<br>C…N = 6.41 Å, *p* = 33.90 D	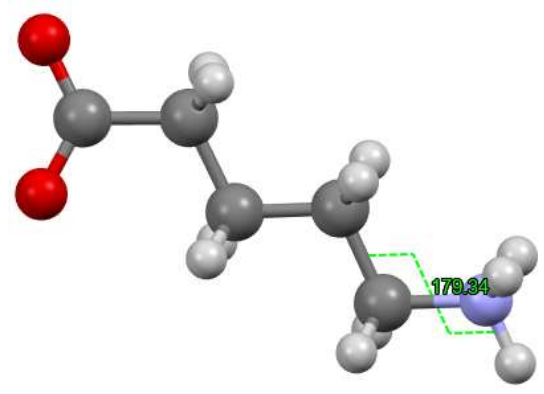<br>C-C-N-H = -61, 59, 179 deg

Table S4 Molecular properties of **canonical forms** of amino acids by HF-MP2 and B3LYP methods.

Basis set def2-TZVPD, in water		Glycine	Glycine	β-alanine	β-alanine
Method		MP2	B3LYP	MP2	B3LYP
1	Energy of HOMO	-163	-260	-251	-157
2	Energy of LUMO	-3.4	38	38	-3.7
3	Energy E^+ in optimized str of L^+	-178332.16	-178101.13	-202727.11	-203000.85
4	Energy E^0 in optimized str of L^0	-178479.28	-178251.76	-202875.12	-203139.68
5	Energy E^- optimized str of L^-	-178504.36	-178276.75	-202897.57	-203169.42
6	Ionization energy, $E_i = E^+ - E^0$, adiabat.	147	151	148	139
7	Electron affinity, $E_{eg} = E^- - E^0$, adiabat.	-25	-25	-22	-30
8	Electronegativity, $\chi_M = (E_i - E_{eg})/2$, adiabat.	86	88	85	84
9	Chemical hardness, $\eta_P = (E_i + E_{eg})/2$, adiabat.	61	63	63	55
10	Dipole moment p / debye	1.754	1.780	1.850	1.930
11	Quadrupole moment Q / ea_0^2, isotropic	-22.71	-22.88	-26.79	-26.62
12	Dipole polarizability α / a_0^3, isotropic	56.81	55.42	71.84	73.86
13	Solvated surface area S / a_0^2	401	400	476	477
14	Solvated volume V / a_0^3	623	622	761	763
15	E_{vib}(ZPE) – zero point energy	49.57	50.01	67.89	67.18
16	Overall $E_{vib}(T^o)$ contribution	51.33	51.73	70.47	69.73
17	$E_{rot} = E_{trs}$ contribution	0.89	0.89	0.89	0.89
18	Inner energy U^o	-178426.17	-178198.25	-202802.87	-203068.07
19	Enthalpy H^o	-178425.58	-178197.65	-202802.28	-203067.47
20	$S_{vib} \cdot T^o$ contribution	2.92	2.86	4.52	5.00
21	$S_{rot} \cdot T^o$ contribution	7.58	7.57	8.01	8.02
22	$S_{trs} \cdot T^o$ contribution	11.59	11.59	11.74	11.74
23	Total entropic term $S \cdot T^o$	22.1	22.0	24.3	24.7
24	Gibbs energy $G^{o,+}$ in optimized str of L^+	-178302.51	-178070.33	-202679.81	-202954.43
25	Gibbs energy $G^{o,0}$ in optimized str of L^0	-178447.67	-178219.67	-202826.54	-203092.22
26	Gibbs energy $G^{o,-}$ in optimized str of L^-	-178475.68	-178247.40	-202851.46	-203124.20
27	Oxidation: $\Delta_r G^o = G^{o,+} - G^{o,0}$	145.2	149.3	146.7	137.8
28	Oxidation potential $E_{abs}^o(L^0/L^+)$ / V	-6.29	-6.48	-6.36	-5.98
29	Reduction: $\Delta_r G^o = G^{o,-} - G^{o,0}$	-28.0	-27.7	-24.9	-32.0
30	Reduction potential $E_{abs}^o(L^0/L^-)$ / V	+1.21	+1.20	+1.08	+1.39

Table S5 Molecular properties of **zwitterionic forms** of amino acids by HF-MP2 and B3LYP methods.

Basis set def2-TZVPD, in water		Glycine	Glycine	β-alanine	β-alanine
Method		MP2	B3LYP	MP2	B3LYP
1	Energy of HOMO	-257	-159	-250	-148
2	Energy of LUMO	39	-0.03	40	-1.6
3	Energy E^+ in optimized str of L^+	-178333.54	-178093.74	-202720.08	-203001.84
4	Energy E^0 in optimized str of L^0	-178483.14	-178256.40	-202875.06	-203140.83
5	Energy E^- optimized str of L^-	-178502.06	-178270.50	-202889.64	-203160.62
6	Ionization energy, $E_i = E^+ - E^0$, adiabat.	163	150	155	139
7	Electron affinity, $E_{eg} = E^- - E^0$, adiabat.	-14.1	-18.9	-14.6	-18.8
8	Electronegativity, $\chi_M = (E_i - E_{eg})/2$, adiabat.	88	84	85	79
9	Chemical hardness, $\eta_P = (E_i + E_{eg})/2$, adiabat.	74	66	70	60
10	Dipole moment p / debye	13.86	13.88	21.60	21.69
11	Quadrupole moment Q / ea_0^2, isotropic	-21.3	-21.2	-24.7	-24.6
12	Dipole polarizability α / a_0^3, isotropic	57.8	58.8	74.8	76.6
13	Solvated surface area S / a_0^2	392	393	477	479
14	Solvated volume V / a_0^3	625	628	763	766
15	E_{vib}(ZPE) – zero point energy	51.1	50.8	69.7	69.0
16	Overall $E_{vib}(T^o)$ contribution	52.7	52.3	71.2	81.4
17	$E_{rot} = E_{trs}$ contribution	0.89	0.89	0.89	0.89
18	Inner energy U^o	-178201.89	-178426.66	-202801.17	-203067.61
19	Enthalpy H^o	-178201.29	-178426.06	-202800.57	-203067.02
20	$S_{vib} \cdot T^o$ contribution	2.7	2.6	4.2	4.4
21	$S_{rot} \cdot T^o$ contribution	7.5	7.5	8.0	8.0
22	$S_{trs} \cdot T^o$ contribution	11.6	11.6	11.7	11.7
23	Total entropic term $S \cdot T^o$	21.8	21.7	23.9	24.1
24	Gibbs energy $G^{o,+}$ in optimized str of L^+	-178301.55	-178061.06	-202670.19	-202952.84
25	Gibbs energy $G^{o,0}$ in optimized str of L^0	-178450.27	-178223.11	-202824.51	-203091.17
26	Gibbs energy $G^{o,-}$ in optimized str of L^-	-178474.53	-178242.91	-202845.11	-203116.66
27	Oxidation: $\Delta_r G^o = G^{o,+} - G^{o,0}$	148.7	162.0	154.3	138.3
28	Oxidation potential $E_{abs}^o(L^0/L^+)$ / V	-7.02	-6.45	-6.69	-6.00
29	Reduction: $\Delta_r G^o = G^{o,-} - G^{o,0}$	-24.3	-19.8	-20.6	-25.5
30	Reduction potential $E_{abs}^o(L^0/L^-)$ / V	0.86	1.05	0.89	1.11

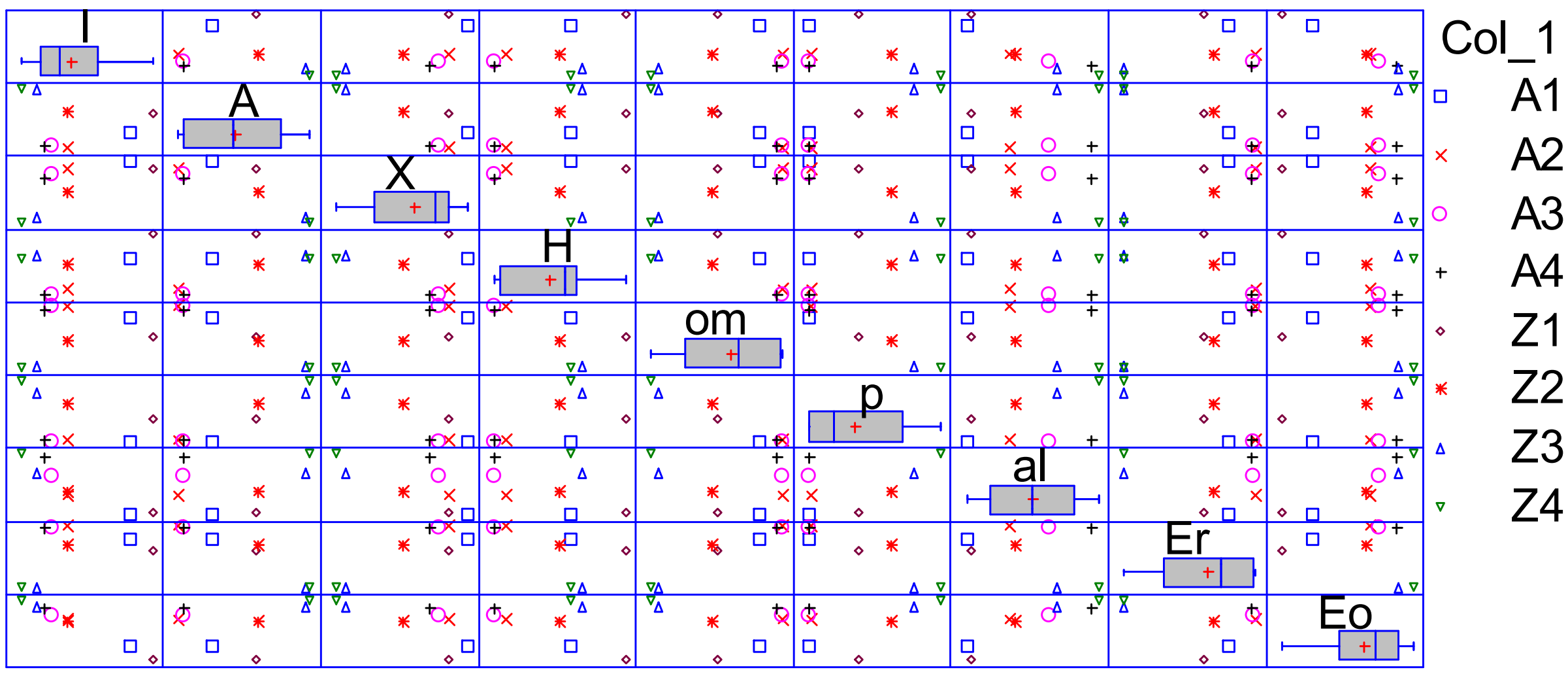

Figure S1 A matrix of scatter points referring to the molecular descriptors of amino acids. Nicknames: I – adiabatic ionization energy, A – electron affinity, X – molecular electronegativity, H – chemical hardness, om – electrophilicity index, p – dipole moment, al – polarizability, Er – reduction potential, Eo – oxidation potential. A1/Z1 – glycine, A2/Z2 – β-alanine, A3/Z3 – GABA, A4/Z4 – DAVA.

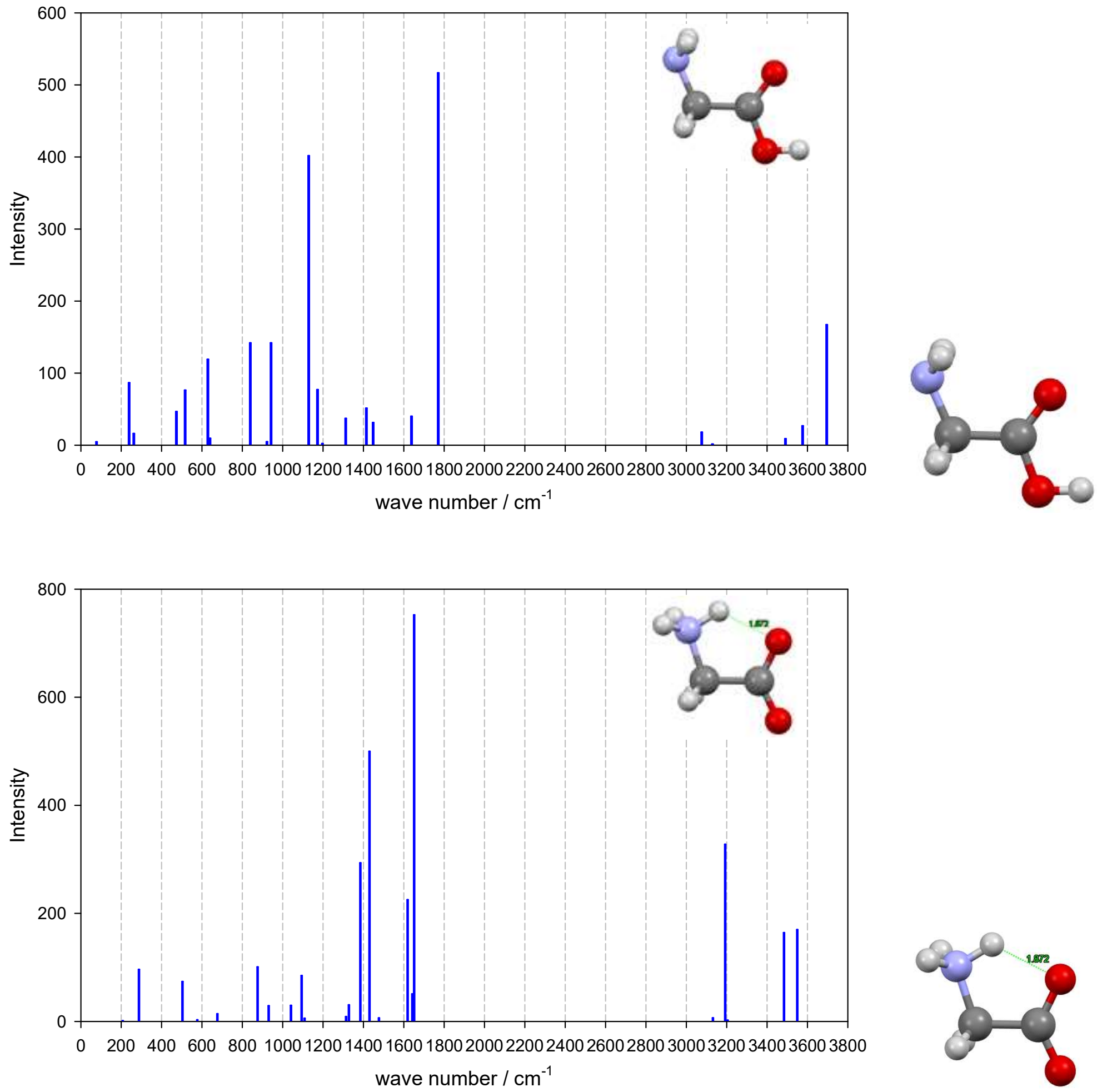

Figure S2 Calculated vibrational spectra (harmonic frequencies) by MP2 in water for glycine, canonical form **1** (top) and **Z**-form (bottom)

Table S6 Calculated (unscaled) harmonic vibrational frequencies [cm^{-1}] in the canonical form **A1** and zwitterionic form **Z** by MP2 and B3LYP methods in water.

β-alanine				GABA		DAVA	
MP2	B3LYP	MP2	B3LYP	B3LYP	B3LYP	B3LYP	B3LYP
**A1**	**A1**	**Z**	**Z**	**A1**	**Z**	**A1**	**Z**
36	9	56	34	37	43	15	50
111	110	115	107	88	75	62	60
180	182	186	183	128	131	99	94
254	240	261	257	136	135	111	121
378	372	351	342	276	220	147	138
459	458	478	474	304	306	224	224
524	510	601	593	347	346	260	245
625	617	693	687	516	529	304	304
636	634	801	793	522	589	436	417
801	794	912	893	623	678	505	522
880	863	946	921	631	762	516	591
914	897	959	941	750	850	616	685
1011	991	1044	1004	849	892	635	749
1076	1053	1120	1110	874	929	745	799
1123	1101	1167	1146	892	974	798	890
1132	1122	1305	1292	1018	993	867	893
1188	1177	1308	1300	1042	1063	878	925
1268	1264	1357	1344	1057	1121	938	993
1302	1297	1397	1372	1090	1147	980	995
1337	1327	1413	1404	1136	1267	1035	1046
1394	1381	1465	1452	1150	1277	1064	1062
1443	1429	1497	1487	1271	1322	1074	1124
1447	1435	1518	1519	1283	1354	1115	1152
1514	1504	1581	1571	1322	1359	1132	1247
1630	1632	1644	1635	1324	1379	1160	1263
1761	1743	1655	1644	1384	1417	1242	1312
3037	2990	3063	3032	1389	1447	1269	1328
3066	3033	3118	3067	1408	1477	1296	1333
3110	3057	3130	3104	1435	1499	1316	1352
3128	3075	3203	3157	1472	1518	1325	1380
3477	3471	3412	3412	1494	1556	1333	1388
3564	3540	3506	3481	1632	1631	1372	1417
3686	3686	3510	3487	1747	1640	1412	1447
				3020	3022	1422	1473
				3025	3034	1435	1488
				3032	3049	1471	1503
				3044	3074	1491	1520
				3058	3084	1509	1545
				3075	3137	1628	1635
				3463	3414	1745	1645
				3530	3484	2956	3010
				3692	3488	3001	3014
						3023	3029

	3026	3040
	3032	3049
	3043	3073
	3052	3085
	3071	3139
	3467	3412
	3536	3481
	3688	3485
